# Convergence of vitamin D and lysophosphatidic acid signaling in stimulating human osteoblast maturation

**DOI:** 10.3389/fphys.2014.00263

**Published:** 2014-07-11

**Authors:** Jason P. Mansell

**Affiliations:** Department of Biological, Biomedical and Analytical Sciences, University of the West of EnglandBristol, UK

**Keywords:** vitamin D, lysophosphatidic acid, osteoblast, alkaline phosphatase, differentiation

Vitamin D is absolutely essential for the provision of a mechanically robust, mineralized bone collagen matrix. Whilst their continues to be a debate over the precise role of this steroid in bone biology it is unequivocal that active vitamin D3 metabolites have direct effects on the control of human osteoblast (hOB) function, matrix calcification and overall bone metabolism. Indeed van de Peppel and van Leeuwen identify many studies supporting a direct effect of the active vitamin D3 metabolite, 1,25-dihydroxyvitamin D3 (1,25D), in hOBs. It is also clear that these cells are receptive to 24,25D, and that the responses observed are a consequence of vitamin D receptor (VDR)-mediated events. Of additional significance are the reports describing cross-talk between 1,25D and other growth factors known to influence hOB activity. These are especially important developments that will help decipher the multiple networks which interconnect with vitamin D in controlling gene expression linked to the regulation of cell growth, bone matrix synthesis and mineralization. The identities of these molecules in turn offer enticing opportunities in developing novel strategies to facilitate 1,25D enhanced bone repair and/or regeneration where it might be needed most, for example at sites of fracture non-unions and at bone biomaterial surfaces to bolster their integration into host tissue.

The cited growth factors known to participate with 1,25D are wide ranging and include local bone mediators, e.g., TGFbeta, the calciotropic hormone PTH and Wnt signaling ligands. Collectively these studies underpin the diversity of signal transduction networks that converge on 1,25D in regulating hOB function. There is however a surprising omission by van de Peppel and van Leeuwen; the simple bioactive glycerophospholipid, lysophosphatidic acid (LPA). This particular lipid is one of only three reported growth factors known to synergistically co-operate with 1,25D in promoting hOB maturation; TGFbeta and EGF are the other two agents. Approximately a decade ago Mansell's group (Gidley et al., 2006) discovered that the ability of 1,25D to stimulate hOB (MG63) alkaline phosphatase (ALP) expression was a consequence of signaling co-operation with serum-borne LPA, as bound to the albumin fraction. In the absence of serum 1,25D had, at best, a trivial influence on ALP expression. Since this initial finding several published works have emerged describing the co-operative effects of LPA and selected LPA receptor agonists with 1,25D/24,25D on hOB formation and maturation, not only in a conventional tissue culture setting but in association with titanium and hydroxyapatite, two widely used bone biomaterials (Blackburn and Mansell, 2012; Lancaster et al., 2014).

It has been known for over two decades that the bulk of serum LPA, the most abundant growth factor in serum, is stably bound to the albumin fraction. Indeed an explanation as to why serum starved fibroblasts accrued enormous quantities of F-actin stress fibers in response to serum was attributed to albumin bound LPA (Ridley and Hall, 1992). Similarly Tigyi and colleagues discovered that the serum-borne component responsible for evoking membrane currents in *Xenopus* oocytes and neurite retraction in PC12 pheochromocytoma cells was LPA tethered to albumin (Tigyi, 2010). Given that 1,25D co-operates with LPA in promoting hOB maturation this raises a potentially important point pertaining to studies investigating the *in vitro* effects of 1,25D, either alone, or in combination with selected agents. It is likely that such studies are inadvertently reporting on signaling cross-talk with LPA associated with whole serum or native, serum albumin. Greater attention should therefore be placed in defining the culture conditions when examining the influence of 1,25D on hOBs; essentially fatty acid-free albumin(s) or charcoal stripped sera are obvious alternatives to eliminate signaling interference from LPA.

The ability of 1,25D to act in concert with LPA in promoting ALP expression is an exciting development in hOB biology. Although it is generally agreed that 1,25D influences the expression of hOB genes synonymous with their transition to a more mature phenotype, the role of 1,25D in the context of ALP expression has yet to be more clearly defined. Despite the clear connection between 1,25D and bone mineralization and the vital role of ALP in this process, ALP, unlike osteocalcin for example, is not identified as a 1,25D responsive gene. This is a particularly intriguing paradox. Whilst there is evidence of a vitamin D response element like-motif in the promoter region of the human ALP gene it is reportedly non-functional. It has been proposed that a role of 1,25D in ALP expression is a consequence of enhanced mRNA stability rather than direct transcriptional control (Orimo and Shimada, 2006; Orimo, 2010). In this regard 1,25D amplifies protein expression by extending the life of the ALP transcript. It is conceivable therefore that the synergistic increases observed for hOB ALP consequent to co-stimulation with 1,25D and LPA, is an enhancement, of an otherwise modest, LPA-induced event. Credence is given to this possibility because LPA (likewise for TGFbeta and EGF), when administered to hOBs, in isolation, stimulates a small increase in ALP (Blackburn and Mansell, 2012). In accordance to the remarks of van de Peppel and van Leeuwen research could now extend to identifying the signaling networks that exist for LPA and 1,25D in hOBs whether they are related directly to hOB fate or to other cell types within the bone microenvironment.

The discovery of signaling co-operation between LPA and 1,25D in the regulation of ALP is an important development given the vital role of this enzyme in bone matrix calcification (Whyte, 2010). Importantly our findings echo an earlier, very significant event in our understanding of signal transduction “cross-coupling” wherein a promitogenic stimulus (e.g., LPA) converges with a pro-differentiating steroid hormone (e.g., 1,25D) response (Schule and Evans, 1991). Identifying LPA as a molecule with the capacity to enhance 1,25D-induced ALP adds another interesting layer that may extend to the processes of bone repair and regeneration; LPA is a product of platelet activation and a recognized mediator of soft tissue healing (Moolenaar et al., 2004). There is potential therefore to consider LPA and related molecules in a bone regenerative context, either in a controlled release format or as coatings to contemporary metal and ceramic devices.

## Conflict of interest statement

The author declares that the research was conducted in the absence of any commercial or financial relationships that could be construed as a potential conflict of interest.
